# Early respiratory interventional therapy combined with antifungal agent for endobronchial cryptococcosis: A case report and literature review.

**DOI:** 10.1097/MD.0000000000037455

**Published:** 2024-03-22

**Authors:** Qinglan Li, Daxiong Wen, Yong Chen, Longfeng Yang, Jing Li, Shaohua Luo

**Affiliations:** a Department of Pulmonary and Critical Care Medicine, Heyuan People’s Hospital, Heyuan, Guangdong, China; b Department of Pathology, Heyuan People’s Hospital, Heyuan, Guangdong, China; c Department of Pulmonary and Critical Care Medicine, Guangdong Provincial People’s Hospital (Guangdong Academy of Medical Sciences), Southern Medical University, Guangzhou, Guangdong, China.

**Keywords:** antifungal agent, case report, early rigid bronchoscopic therapy, endobronchial cryptococcosis, literature review

## Abstract

**Rationale:**

Cryptococcosis presenting as endobronchial obstruction and lung collapse is an extremely rare occurrence. While these patients were treated with antifungal agents, unfortunately, half of them showed a suboptimal response.

**Patient concerns:**

A 45-year-old immunocompetent male was admitted to the hospital due to a cough, yellow phlegm, and dyspnea persisting for 5 months. Chest computer tomography revealed a mass in the right main bronchus accompanied by right lower lobe atelectasis.

**Diagnoses:**

Endobronchial cryptococcosis presenting as endobronchial obstruction and lung collapse.

**Interventions:**

Early rigid bronchoscopic therapy was performed to resect endobronchial obstruction, which combined with antifungal agent.

**Outcomes:**

The patient recovered well with completely clinical and radiologic resolution at 1 year follow-up.

**Lessons:**

This case provides a good example of successful utilization of the early respiratory interventional therapy combined with antifungal agent in obstructive endobronchial cryptococcosis.

## 1. Introduction

Cryptococcosis is an infection caused by the yeast-like fungus *Cryptococcus*. It is generally associated with exposure to contaminated pigeon droppings. Pulmonary cryptococcosis usually presents as a single mass or multiple nodules, rarely as endobronchial lesions, and even more rarely causing atelectasis. In published articles, primary pulmonary cryptococcosis leading to obstructive atelectasis caused by an airway mass has generally been treated initially with antifungal therapy. Respiratory interventional therapy or surgical lung resection was used when the antifungal therapy proved ineffective. However, to the best of our knowledge, a combination of early respiratory interventional therapy with antifungal therapy is rarely employed in cases of obstructive endobronchial cryptococcosis. In this report, we present a rare case of pulmonary cryptococcosis manifested as atelectasis due to an endobronchial mass in an immunocompetent patient. The patient underwent bronchoscopy with mass resection, and subsequently was diagnosed with endobronchial cryptococcosis. Antifungal treatment using fluconazole was initiated. Additionally, we conducted a literature review on the clinical features and treatment of pulmonary cryptococcal infection-induced atelectasis.

## 2. Case presentation

A 45-year-old man was admitted to the hospital due to a cough, yellow phlegm, and dyspnea persisting for 5 months. He denied experiencing hemoptysis, chills, fever, night sweats, chest tightness, chest pain, dizziness, headache, nausea, vomiting, abdominal pain, diarrhea, dysuria, or weight loss. A chest computer tomography (CT) conducted 4 days before admission revealed a mass in the right main bronchus accompanied by right lower lobe atelectasis. He was admitted for further diagnosis and treatment. Physical examination revealed diminished breathing sounds in the right lower lung, while the rest of the examination was normal. The patient had a history of smoking 20 cigarettes per day for 28 years but no allergies, illegal drug use, current medication, or pets. However, after the diagnosis, it was found that the patient had visited a pigeon breeding place once a week for approximately 5 years.

**Figure 1.**
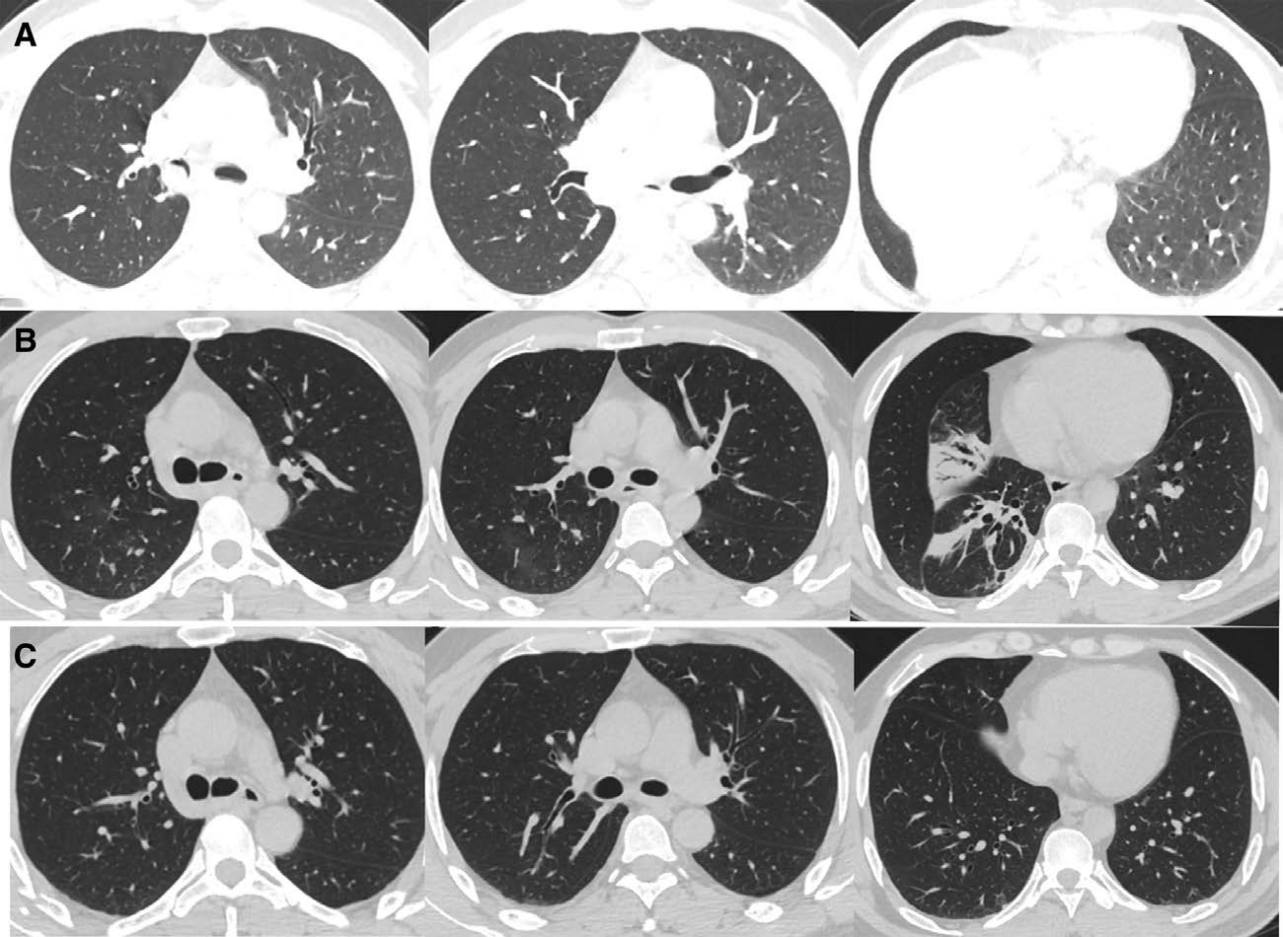
Computerized tomography (CT) scan. (A) Showing a mass in the right main bronchus and right intermediate bronchus, along with atelectasis in the right lower lobe; (B) demonstrating the disappearance of the mass in the right main bronchus and obvious lung recruitment. (C) Illustrating the complete disappearance of the mass in the right main bronchus and complete lung recruitment.

**Figure 2.**
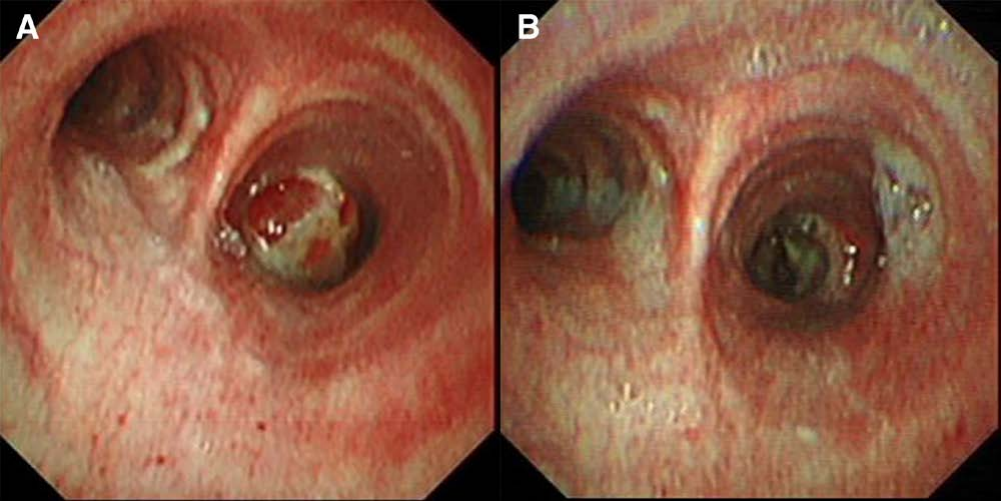
Bronchoscopy examination. (A) Flexible bronchoscopy before rigid bronchoscopy: showing a mass in the right main bronchus that caused occlusion; (B) flexible bronchoscopy after 3 d of rigid bronchoscopy: demonstrating successful removal of the mass from the right main bronchus.

Blood assay results showed a white blood cell count of 6.99 × 10^9^/L, lymphocyte count of 1.19 × 10^9^/L, hemoglobin concentration of 165.00 g/L, and platelet count of 288.00 × 10^9^/L. Tests for routine urine, routine stool, renal function, liver function, blood sugar, coagulation function, and tumor markers all returned normal results. Sputum smears for fungi or acid-fast bacilli were negative, and no pathogens were detected in sputum and blood samples. Enhanced chest CT conducted at our hospital revealed the following findings: A tumor-like mass in the lower segment of the right main bronchus and right intermediate bronchus, along with atelectasis in the right lower lobe; Multiple nodules in the upper lobes of both lungs and the lower lobe of the left lung (Fig. 1A). Flexible bronchoscopy revealed a white mass in the right main bronchus, occluding the lumen (Fig. 2A). Histological examination of the specimen revealed chronic mucosal ulceration with granuloma formation. However, no culture was performed for pathogen identification.

The patient chest CT indicated obstructive atelectasis caused by a mass, but the preliminary pathological biopsy did not definitively support a tumor diagnosis. This uncertainty might be related to superficial sampling, although neoplastic lesions cannot be completely ruled out. Therefore, obtaining a larger tissue sample for pathological examination was recommended to confirm the diagnosis and relieve airway obstruction using rigid bronchoscopy. During the operation, a white mass was observed, which was loosely attached to the bronchial wall, and there was minimal bleeding when the mass was removed with forceps. Histological examination of the mass revealed chronic suppurative inflammation and granuloma formation, many multinucleated giant cells, and circular refraction in the cytoplasm samples, suggesting *Cryptococcus neoformans* infection. The presence of *C neoformans* was confirmed in alveolar lavage fluid through metagenomic next-generation sequencing. Meanwhile, the serum *Cryptococcus* capsular antigen test yielded a positive result.

The results of cerebrospinal fluid examination and brain magnetic resonance imaging enhanced scan showed no signs of intracranial infection, so he was treated with fluconazole 400 mg/d intravenous infusion. The patient dyspnea improved significantly after the operation, and chest CT 3 days later showed obvious recruitment of the lungs (Fig. 1B). Flexible bronchoscopy showed patchy lesions in the bronchi (Fig. 2B).

After discharge, the patient continued to take fluconazole 300 mg per day for 6 months. During the 1-year follow-up, the patient symptoms completely disappeared, and a subsequent CT scan showed complete lung recruitment with the lesions no longer present (Fig. 1C).

## 3. Discussion and conclusions

Cryptococcosis is an invasive fungal infection caused by *Cryptococcus*. Previously considered a rare disease, an increasing number of cases of cryptococcosis are being diagnosed as detection techniques improve. Most cryptococcosis is caused by 2 species *C neoformans* and *Cryptococcus gattii*.^[1]^ Risk factors for cryptococcosis include HIV infection, organ transplantation, the use of steroids or immunosuppressants, malignancy, sarcoidosis, idiopathic CD4 + lymphopenia, rheumatic disease, and anti-GM-CSF autoantibodies.^[2]^ However, cryptococcosis also frequently occurs in apparently healthy hosts. In general, pulmonary cryptococcosis is difficult to diagnose because symptoms and radiological findings are nonspecific and can vary according to the patient immune status.^[3]^ The development of endobronchial lesions is a rare manifestation of pulmonary fungal infection. A review of the literature by Karnak et al^[4]^ found that the majority of these cases were associated with *Aspergillus* infections. Endobronchial infections are caused by *Coccidioides immitis*, Zygomycetes, Candida species, *C neoformans*, and *Histoplasma capsulatum* uncommonly. The pathogenesis of endobronchial cryptococcal infection has not been fully clarified. Various sources of this pulmonary infection may include direct engraftment of yeast components into the bronchi from adjacent parenchymal lesions, direct airway infiltration from adjacent intrathoracic lymph nodes, erosion and protrusion of intrathoracic lymph nodes into the bronchi, hematogenous dissemination, and extension to the peribronchial area by lymphatic drainage.^[5]^ It is also possible that *Cryptococcus* directly invades the bronchi. It has been reported that endobronchial cryptococcal infection only exists in the bronchi,^[6]^ suggesting that the pathogen may directly invade the bronchi. Sixty-three percent of cryptococcal endobronchial infections described in the literature occurred in immunocompetent patients.^[7]^ Therefore, the immune status of patients does not seem to affect the development of such endobronchial lesions. Bronchoscopic findings of cryptococcal endobronchial infection include white or red plaques, ulcers, polyps, nodules, lobes, hemorrhages, raised or space-occupying lesions. The relationship to the bronchial lumen can be obstructive (associated with atelectasis) or nonobstructive (manifested by various mucosal irregularities without atelectasis).

A review of the literature revealed 7 reports of obstructive endobronchial lesions.^[8–14]^ The bronchoscopic feature and associated imaging findings and treatment options for these lesions are summarized in Table 1. Notably, all the obstructive endobronchial cryptococcosis patients reported were immunocompetent. Initially, these cases were treated with antifungal drugs, but 4 of them showed poor response to antifungal therapy, leading to subsequent surgical pneumonectomy or respiratory intervention to remove the airway mass.^[8,15–17]^ Among these cases, 3 patients opted for fluconazole treatment, while 1 patient received amphotericin B alone. Of these, 3 patients achieved complete clinical and radiographical improvement through the combination of amphotericin B with flucytosine. The literature has indicated that the combined treatment of amphotericin with flucytosine yields stronger early fungicidal activity compared to amphotericin B alone.^[18,19]^ Based on this evidence, we believe that amphotericin combined with flucytosine may be more appropriate for obstructive cryptococcal endobronchial lesions.

**Table 1:** Reported cases of obstructive endobronchial cryptococcosis and treatment course.

Ref	Age	Sex	Tobacco history	Underlying disease	Species	Radiographic finding	Endobronchial abnormality	Medical therapy	Response to medical therapy	Subsequent management
Long RF, 1972^[8]^	36	M	30 cigarettes·d-1 for 20 yr	(−)	*Cryptococcus neoformans*	RUL atelectasis	Gelatinous mass	Ampho-B for 30 d	No response	Right pneumonectomy
Town GI 1985^[9]^	26	M	?	(−)	*C neoformans*	RML atelectasis	Hemorrhagic carinal mass	Ampho-B and 5-FC for 4.5 weeks	Complete clinical and radiologic resolution	None
Carter EA 1992^[10]^	65	M	?	(−)	*Cryptococcus gattii*	Left lung collapse	Endobronchial mass	Ampho-B and 5-FC, followed by Fluconazole	Complete clinical and radiologic resolution	None
Mahida P 1996^[11]^	43	M	20 cigarettes·d-1	(−)	*C neoformans*	RML and RLL atelectasis	White lobulated endobronchial mass	Ampho-B/5-FC for 2 mo, followed by itraconazole for 7 mo	Complete clinical and radiologic resolution	None
Chang Y-S, 2005^[12]^	33	M	?	(−)	*C neoformans*	LUL atelectasis	Whitish mass	Fluconazole for 8 weeks	No response	LUL lobectomy
Artinian V, 2010^[13]^	46	M	60 cigarettes·d-1 for 30 yr	(−)	*C neoformans*	RUL atelectasis	Large glistening mass	Fluconazole for 4 weeks	Progression	Bronchoscopy resection and the placement of stent.
Huynh J, 2020^[14]^	11	F	(−)	(−)	*C gattii*	LLL consolidate, LUL collapse	Thick gelatinous mass	Fluconazole for 18 d	Progression	Bronchoscopy resection
Present case	45	M	20 cigarettes·d-1 for 30 yr	(−)	*C neoformans*	RLL consolidate, RUL collapse	Irregular White mass	Bronchoscopy resection, And Fluconazole for 6 mo	Complete clinical and radiologic resolution	None

5-FC = 5-fluorocytosine, Ampho-B = indicates amphotericin B, F = indicates female, LLL = left lower lobe, LUL = left upper lobe, M = indicates male, Ref = reference, RLL = right lower lobe, RML = right middle lobe, RUL = right upper lobe, RUL = right upper lobe.

However, in the case we present here, the patient underwent rigid bronchoscopy before receiving antifungal drugs. During the procedure, we observed that the mass was not firmly adhered to the airway wall, and we were able to remove it relatively intact. Subsequent biopsy identified cryptococcal infection, and the patient was subsequently treated with fluconazole. Follow-up assessments demonstrated significant clinical and imaging improvements. After the removal of the airway mass through bronchoscopy, the patient symptoms were relieved, CT scans showed a reduction in lung lesions, and the patient condition improved, indicating successful treatment with fluconazole.

Solitary cryptococcal pulmonary infections in immunocompetent patients may resolve spontaneously without specific treatment.^[20]^ Monitoring alone has been suggested as sufficient for such patients, and antifungal therapy may not be necessary.^[15,20,21]^ However, it is advisable to consider antifungal therapy for cases of cryptococcosis exhibiting persistent or severe symptoms, or extensive lesions evident on chest X-rays.^[20]^ Endobronchial cryptococcosis is almost always accompanied by pulmonary lesions (except in 2 cases^[22,23]^). Considering the possible pathogenesis, a study reported a case in which non-complete obstructive bronchial cryptococcal lesions progressed to complete obstruction.^[24]^ Therefore, it is important to recognize that obstructive endobronchial cryptococcosis can be a progressive disease, necessitating prompt administration of antifungal therapy. Nonetheless, the antifungal response is generally slow, and approximately half of patients with obstructive bronchial cryptococcosis do not respond effectively to antifungal therapy. This suggests that, for patients with significant airway obstruction due to endobronchial cryptococcosis, timely airway intervention measures should be considered.

This report highlights the potential effectiveness of early rigid bronchoscopy in managing endobronchial infections that result in occlusive disease or atelectasis. To the best of our knowledge, this is the first documented case of early rigid bronchoscopy combined with antifungal agents for treating obstructive endobronchial cryptococcosis. Respiratory interventions successfully reduced the fungal burden and maintained airway patency. As a result, the patient experienced improved symptoms, lung function, and resumed daily activities.

Some may question the necessity of early endobronchial intervention, instead suggesting antifungal therapy initially, with respiratory intervention or surgical resection recommended if medical treatment proves ineffective. However, we hold the view that opting for early respiratory intervention is a rational choice. Firstly, it important to note that nearly half of patients with obstructive endobronchial cryptococcosis exhibit poor responses to antifungal medications. Secondly, prolonged atelectasis presents challenges in achieving lung recruitment, potentially leading to permanent lung collapse and compromised lung function, or even eventual lung parenchymal infection and fibrosis.^[16]^ Moreover, addressing the underlying cause of atelectasis is paramount to its treatment. Bronchoscopy, with its diagnostic and therapeutic capabilities, proves valuable in managing atelectasis. Flexible bronchoscopy aids in distinguishing between intrinsic obstruction and extrinsic compression, while rigid bronchoscopy provides better insight into the nature of intrinsic obstructive lesions and offers a means for endotracheal therapy.^[17]^ In this particular case, the patient experienced only minimal bloody sputum within 3 days following the operation, indicating the safety and effectiveness of utilizing rigid bronchoscopy.

Given the limited understanding surrounding severe airway obstruction resulting from endobronchial cryptococcosis, a consensus on the management of obstructive endobronchial cryptococcosis has yet to be established. However, this case serves as practical evidence supporting the early implementation of respiratory interventional therapy in cases of obstructive endobronchial cryptococcosis.

## Author contributions

**Conceptualization:** Qinglan Li, Shaohua Luo.

**Funding acquisition:** Qinglan Li.

**Investigation:** Qinglan Li, Daxiong Wen, Yong Chen, Longfeng Yang, Jing Li, Shaohua Luo.

**Supervision:** Jing Li.

**Validation:** Qinglan Li, Daxiong Wen, Yong Chen, Longfeng Yang, Jing Li, Shaohua Luo.

**Writing – original draft:** Qinglan Li.

**Writing – review & editing:** Qinglan Li, Shaohua Luo.
